# Epidemiology of sports injuries on collegiate athletes at a single center

**DOI:** 10.1590/1413-78522014220601007

**Published:** 2014

**Authors:** Bruno Berbert Rosa, André Marangoni Asperti, Camilo Partezani Helito, Marco Kawamura Demange, Tiago Lazzaretti Fernandes, Arnaldo José Hernandez

**Affiliations:** 1. Universidade de São Paulo, Faculdade de Medicina, Institute of Orthopedics and Traumatology, São Paulo, SP, Brasil, Institute of Orthopedics and Traumatology, Faculdade de Medicina da Universidade de São Paulo (FMUSP), São Paulo, SP, Brasil

**Keywords:** Athletic injuries, Knee, Ankle, Epidemiology

## Abstract

**OBJECTIVE:**

To evaluate the incidence of sports injuries in college athletes from the same institution from 1993 to 2013.

**METHODS:**

Athletes from 13 modalities were interviewed about the presence and type of injury, type of treatment and time of withdrawal, based on the questionnaire "Injury Surveillance System" (ISS). Data were analyzed with graphs and tables of injury prevalence by gender, age, site of injury and modality. We also analyzed the average time of withdrawal of athletes, returning to sports practice and new lesions.

**RESULTS:**

It was observed that 49.91% of the athletes showed some type of injury, with similar incidence between genders; the most frequent injuries were the anterior cruciate ligament (ACL) and the ankle sprain; the average withdrawal time was 11 weeks. ACL was the injury with greater impact on college sports career, especially given the time of withdrawal.

**CONCLUSION:**

The most frequent injury, ACL, occurred most frequently in indoor sports such as handball and volleyball and had the highest number of cases treated with surgery and a longer average withdrawal time. More studies are needed to create a larger database in order to schedule preventive measures for amateur athletes.** Level IV of Evidence, Epidemiological Study.**

## INTRODUCTION

University sports in Brazil do not have the expression observed in other countries such as the USA,^1^^,^ 2 in which the participation of athletes in universities is linked to scholarships and the professionalization of the sports career is a reality.

The Faculdade de Medicina da Universidade de São Paulo (FMUSP), São Paulo, SP, Brazil, has an athletic association (AAAOC) structured for the practice of intercollegiate sports, including skilled professionals as coaches and trainers. The frequency of training is daily and students are exposed to injuries such as ligament ruptures and fractures.^1^^-^ 3 One of the risk factors for such events is the fact that most of these athletes did not previously practiced sports with the above mentioned frequency, often not being adequately fit.^4^^,^ 5

More than 100 college students a year enter in the Athletic Association and these athletes train on average 10 hours per week, in addition to matches. They participate in local, state and national competitions by, on average, 6 to 10 years, while in medical school.

In the USA, there is a control of the types and distribution of injuries in college sports. At the "American National College Association" (NCAA), the control is done since 1982.^2^^,^ 6 Such information is useful to promote prevention strategies in sport, such as specific strengthening training, muscle strengthening and physiotherapy.^7^^-^ 13 In Brazil, up to now, there are no studies showing the profile of injuries at university sports, hindering prevention strategies. Therefore, the aim of this study was to assess the epidemiological evolution of these injuries in the university environment of FMUSP in the last 20 years and identify key predictors of injuries.

## METHODS

Eight hundred and thirty seven college athletes, of both genders, who participated in sports in the last 20 years at FMUSP were selected.

The sports categories included in this research were: futsal, handball, women's basketball and volleyball, softball, athletics, soccer, rugby, water polo, martial arts (judo and karate), handball, basketball and volleyball.

We used a brief version of the questionnaire "Injury Surveillance System" (ISS)^2^ in which information from the site of injury, mechanism of injury, year of injury and sport practiced were collected.

The athletes were also asked regarding the type of treatment, surgical or conservative and the time of withdrawal from sports. The withdrawal time was calculated in weeks. Respondents were asked about the use of information and all of them authorized the publication of the data.

### Statistical analysis 

Demographic analysis with graphs and tables of injury prevalence by gender, age, site of injury and sport were held. We also analyzed the average time of withdrawal of athletes, whether they returned to sports practice and showed new injuries.

## RESULTS

**Figure 1:**
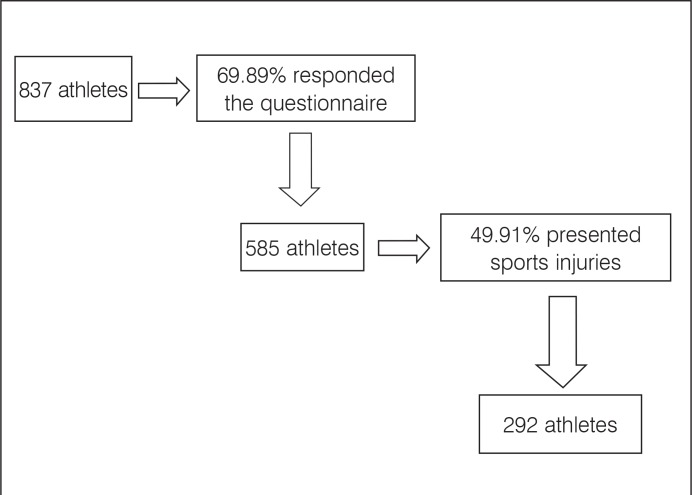
Flowchart of cases included in the study.

Figure 1 shows the flowchart of the cases included in the survey used for the statistical analysis, where 837 athletes were surveyed, 69.89% answered the questionnaire (585) and 49.91% had sports injuries (292).

Among men, 49.1% (165) of the athletes had injuries. The percentage among women was 51% (127), which shows the similarity in injury incidence between both genders.

The most common injury was to the anterior cruciate ligament (ACL), followed by the ankle ligament injury, which together represent 25% of cases. Only severe ligament injuries of the ankle were included, with time of withdrawal greater than 14 days. Injuries in descending order (i.e.: fractures of fingers, stress fracture of the tibia and glenohumeral dislocation) are presented in Table 1.

**Table 1:** Incidence of most frequent injuries.

Injury	Frequency (n)	Incidence
Anterior cruciate ligament	37	12.7%
Ankle ligament injury	36	12.3%
Fractures of fingers	26	8.9%
Stress fracture of the tibia	18	6.2%
Glenohumeral dislocation	16	4.8%
Others	161	48.6%

n = number of events.

Among the categories studied, the ACL injury was more prevalent in women's handball, followed by men's volleyball. The ankle ligament injury was more common in volleyball, being more frequent in women than in men, as shown in Table 2.

**Table 2:** Incidence of anterior cruciate ligament and ankle ligament injury at sports.

Sport	ACL injury (n)	Ankle ligament injury (n)
Women's Handball	16% (8)	8% (4)
Men's Volleyball	12.5% (4)	12.5% (4)
Women's Volleyball	9.09% (4)	13.6% (6)
Soccer	6.25% (5)	6.25% (5)
Women's basketball	4.41% (3)	5.88% (4)
Men's Handball	4.61% (3)	6.15% (4)

n = number of events.

The ACL injury alone occurred in 37 of the 585 athletes of the study, or 6.3% of the total. The incidence in women was slightly higher, with 18 cases in 249 of athletes or 7.2%. In men, there were 19 injuries in 336 athletes or 5.6%.

Analyzing the site of injury, 55.8% of injuries were in the lower limbs, especially in athletics and football (88.6% and 78.8% respectively). Injuries to the upper limb, although less prevalent in general, were more common in water polo (100%) and softball (75%). Table 3 shows the division by site and type of injury.

**Table 3:** Injury location and sports category.

Category	Upper limbs (n)
Water polo	100% (6)
*Softball*	75% (9)
Martial Arts	73.7% (14)
Rugby	68.4% (13)
Men's Basketball	57.9% (11)
**Category**	**Lower limbs (n)**
Athletics	88.6% (31)
Soccer	78.8% (26)
Women's basketball	77.8% (14)
Women's Futsal	76% (19)
Women's Handball	66.7% (16)

n = number of events.

Regarding the type of treatment, the vast majority of cases (72.9%) were treated conservatively. A sport in which injuries resulted in greater number of surgical treatments was men's volleyball, with 56.3% of cases. In all other categories, conservative treatment was more frequent than surgical. In athletics, only 5.3% of the cases resulted in surgery.

**Figure 2:**
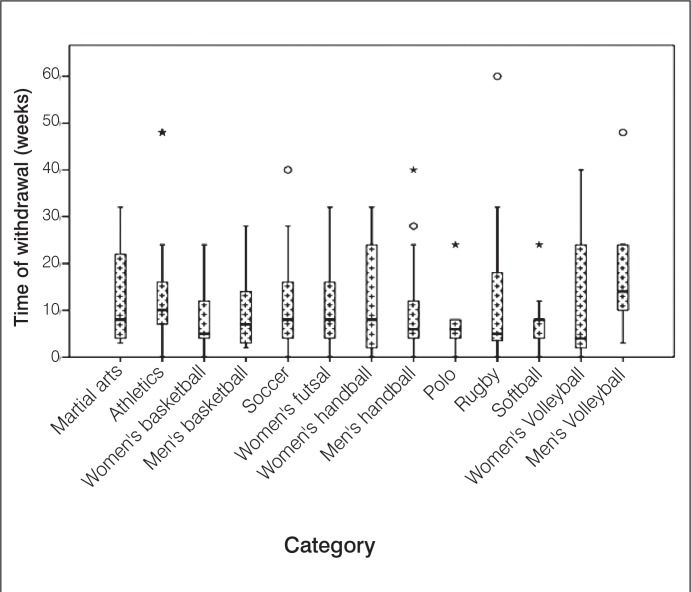
Withdrawal from sports in weeks.

**Figure 3:**
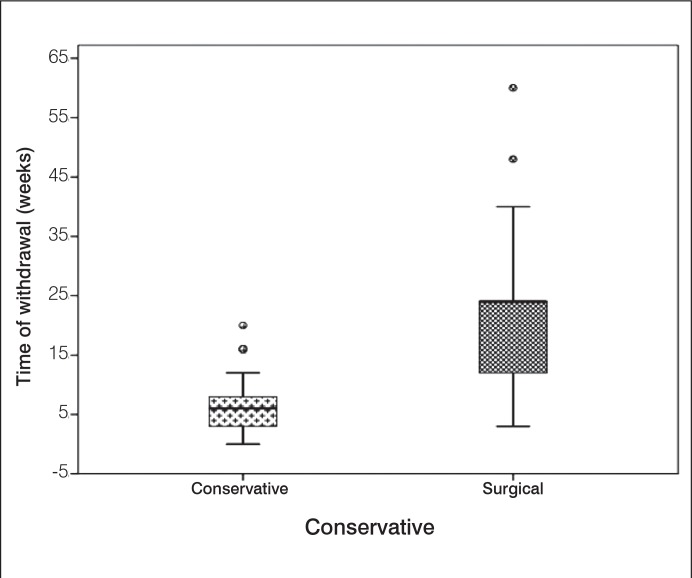
Withdrawal according to type of treatment.

The sport with the longest withdrawal time was the men's volleyball, with an average of 16 weeks. In general, the average withdrawal time was 11 weeks, including conservative and surgical treatments. There is a direct relationship between the time of withdrawal and the type of treatment and in injuries treated surgically, the time off was higher, approximately 21 weeks. Nine cases left sports practice after injury. (Figures 2 and 3)

## DISCUSSION

The study shows a representative demographic pattern of injuries in the national university sports. The most important result of this study was the high incidence of serious injuries in college athletes, with time off greater than 10 days.^2^^,^ 14 Half of the athletes studied had at least one injury with this feature. This number indicates that college sports practice has a significant impact on the lives of college students, which reinforces the need to schedule preventive measures.

The fact that many athletes did not perform sports activities or performed non-competitive sports activities before entering college can be a risk factor for new injuries, as these athletes do not have adequate pre muscular work nor education and training base to support the high training frequency.^4^

The results are in agreement with the studies of Van Mechelen *et al*.^14^ and Hootman *et al*.^5^, in which the ACL injury and ligament injury of the ankle are the most frequent injuries in college sports. The relationship with the most affected sports is also true, and volleyball is the sport category in which these lesions are more frequentes.^15^

The injury with greater impact on the lives of the college athletes remains the ACL, with the largest number of surgical treatments and time off. Van Mechelen *et al*.^14^ and Hootman *et al*.^5^ also described a higher prevalence of these injuries in the female audience. Systematic reviews and meta-analyzes also demonstrate higher prevalence in the feminine gender.^15^^,^ 16

The results presented in specific sports categories such as athletics and water polo show that the type of training and exercise are directly related to injuries. In water polo, for example, there is a high prevalence of injuries in the upper limbs due to pitch and dispute for the ball with the hands. However, in athletics, because of the high intensity of training, there is a high prevalence of stress fractures. In court sports, due to jumping and cutting movements, there is a high prevalence of sprains in the lower limbs.

By not having a basis of previous data on the epidemiology of injuries in college sports, it has been difficult defining prevention strategies for each sports category.^1^ We observed that the creation of a national system for data collection, such as in the USA,^2^^,^ 17 shall facilitate the design of preventive measures in order to reduce the incidence of injuries and the withdrawal time of these athletes.

A methodological limitation of this study is its retrospective nature, in which that in order to answer the questionnaire participants rely on their memories, which can lead to recollection bias. Minor injuries can be forgotten and not mentioned.

## CONCLUSION

Future studies are being conducted in a prospective manner in order to improve the quality of information and create a solid database. The clinical relevance of this study is, therefore, to present demographic information of major injuries in college sports, allowing proposing strategies for injury prevention and health promotion.
